# Proteins of the Lectin Pathway of complement activation at the site of injury in subarachnoid hemorrhage compared with peripheral blood

**DOI:** 10.1002/brb3.1728

**Published:** 2020-06-21

**Authors:** Thorkil Anker‐Møller, Anne‐Mette Hvas, Niels Sunde, Steffen Thiel, Anne Troldborg

**Affiliations:** ^1^ Thrombosis & Haemostasis Research Unit Department of Clinical Biochemistry Aarhus University Hospital Aarhus Denmark; ^2^ Department of Neurosurgery Aarhus University Hospital Aarhus Denmark; ^3^ Department of Biomedicine Aarhus University Aarhus Denmark; ^4^ Department of Rheumatology Aarhus University Hospital Aarhus Denmark

**Keywords:** complement system, lectin pathway, subarachnoid hemorrhage

## Abstract

**Background:**

A subarachnoid hemorrhage (SAH) is a debilitating stroke. Activation of the lectin pathway (LP) of the complement system in SAH patients could worsen the prognosis; however, conflicting results have been reported. This potentially reflects that pathological changes at the site of injury are not reflected in peripheral blood.

**Aims of the study:**

To measure the concentration of LP proteins in blood from the site of brain injury compared with peripheral blood in SAH patients, and to determine the concentration of LP proteins in cerebrospinal fluid (CSF).

**Methods:**

We included 11 SAH patients undergoing aneurysm clipping or external ventricular drainage. Blood was collected from the site of injury and from a peripheral artery and/or CSF simultaneously. LP proteins were measured using time‐resolved immunofluorometric assays.

**Results:**

In all patients, the cerebral blood concentration of mannan‐binding lectin, collectin liver‐1 and collectin kidney‐1, and mannan‐associated serine proteases 1 and 2 were lower than in peripheral blood. The LP proteins were almost undetectable in CSF.

**Conclusion:**

Lectin pathway protein concentrations measured in peripheral blood do not always reflect changes at the site of injury. For some proteins, more information could be obtained in blood from the site of injury when investigating pathogenic mechanisms.

## Graphical abstract

A subarachnoid hemorrhage is a debilitating stroke and activation of the lectin pathway could partially explain the poor prognosis. Conflicting results have been reported regarding the activation of the lectin pathway, which potentially reflects that changes at the site of injury are not reflected in peripheral blood. We found that the concentrations of lectin pathway proteins in peripheral blood do not always reflect changes at the site of injury.

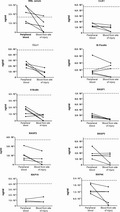

## 1 INTRODUCTION

A subarachnoid hemorrhage (SAH) is a debilitating stroke that often strikes at a young age (Hop, Rinkel, Algra, & van Gijn, 1997) with a median case‐fatality rate between 27% and 44%. (Nieuwkamp et al., 2009) The high mortality and morbidity is caused both by the primary injury of the hemorrhage itself, and by the delayed injury from cerebral vasospasms, cerebral ischemia, and inflammatory reactions (Lucke‐Wold, Logsdon, & Manoranjan, 2016). Several studies have investigated the underlying causes of inflammation following SAH and demonstrated involvement of the complement system (Llull et al., 2017).

The complement system is activated through three distinct pathways: the classical, alternative, and lectin pathway (LP) (Merle & Church, 2015) Particularly, the LP has received increasing focus during recent years because of the potentially pathological role in cerebral ischemia (Llull et al., 2017). The LP is triggered by patterns of carbohydrates or acetyl groups on pathogen surfaces and host cells with altered glycosylation patterns (Baines & Brodsky, 2017). The pattern recognition molecules of the LP that recognize such altered patterns include mannan‐binding lectin (MBL); H‐, M‐, and L‐ficolin (Merle & Church, 2015); collectin liver‐1 (CL‐L1, also named collectin 10) (Ohtani et al., 1999); and collectin kidney‐1 (CL‐K1, also named collectin 11). Serine proteases are attached to the recognition molecules and become activated upon binding of the pattern recognition molecules to targets. These include MBL‐associated serine proteases 1, 2, and 3 (MASP‐1, MASP‐2, and MASP‐3). In addition, the associated proteins, with an as of yet unclear role, MBL‐associated protein of 44 and 19 kDa (MAp44 and MAp19), are also found attached to the recognition molecules of the LP (Kjaer, Thiel, & Andersen, 2013; Merle & Church, 2015).

Most studies demonstrating changes in the complement system following SAH use peripheral blood samples or cerebrospinal fluid (CSF) as surrogate markers of the local cerebral changes (Llull et al., 2017; Mack et al., 2007; Pradilla, Chaichana, Hoang, Huang, & Tamargo, 2010; Zanier et al., 2014). However, it is not known whether local changes at the site of injury are reflected systemically in the blood.

The aim of this exploratory study was firstly to measure the concentration of LP proteins at the site of injury compared with peripheral blood in SAH patients and secondly to measure the concentration of LP proteins in SAH patients in CSF compared with peripheral blood.

## 2 METHODS

### 2.1 Study populations

We performed a prospective explorative cohort study including SAH patients undergoing aneurysm clipping or external ventricular drainage. The patients were included at Department of Neurosurgery, Aarhus University Hospital, Denmark, from November 2016 to August 2017. Inclusion criteria were as follows: spontaneous or traumatic SAH diagnosed by computed tomography (CT) or magnetic resonance imaging (MRI), age older than 18 years. Exclusion criteria were as follows: pregnancy, active cancer, or chemotherapy within the past 3 months. A written informed consent was obtained from all legally competent patients, or from next of kin in legally incompetent patients.

Results of LP protein measurements in serum from a group of blood donors previously described (Troldborg et al., 2017) were used as a reference for measurements in the present study.

The Central Denmark Region Committees on Health Research Ethics and the Danish Data Protection Agency approved the study (case no. 1‐16‐02‐504‐16).

### 2.2 Specimen sampling

All samples were collected in 4‐ml serum tubes. During clipping procedures, a cerebral blood sample from the site of injury was obtained with a syringe and needle and extracted to a 4‐ml serum tube. Simultaneously, a peripheral serum sample from the patient's arterial catheter was extracted. During external ventricular drain procedures, a CSF sample was obtained from the drain immediately after being placed in the cerebral ventricles or from a lumbar puncture during the aneurysm coiling operation. Samples rested 25 min before centrifugation at 3,000 ***g*** for 25 min. Serum was collected, aliquoted, and stored at −80°C until batch analysis.

### 2.3 Laboratory analyses

The serum concentrations of LP proteins were measured at the Department of Biomedicine, Aarhus University, Denmark, using an in‐house time‐resolved immunofluorometric assays (TRIFMA) as previously described. (Troldborg et al., 2017) We tested for the following LP proteins: the recognition molecules MBL, H‐ficolin, M‐ficolin, L‐ficolin, CL‐L1, CL‐K1, and the associated proteins MASP‐1, MASP‐2, MASP‐3, MAp44, and MAp19. All samples were performed in duplicate, and means of the duplicate values were used for statistical analyses. Internal controls were applied for each plate. If the interassay coefficient of variation exceeded 15%, the measurements were repeated.

## 3 RESULTS

Eleven patients were included: nine women and two men with a median age of 51 years (range 39–73). We obtained peripheral and cerebral blood samples from four patients. From six patients, we obtained samples from peripheral blood and CSF, and from one patient, we managed to get both peripheral blood, cerebral blood, and a CFS sample (Table 1).

**Table 1:** Samples available from included patients

Patient	Peripheral serum sample	Cerebral serum sample	CSF
1	x	x	
2	x	x	
3	x	x	
4	x	x	
5	x	x	x
6	x		x
7	x		x
8	x		x
9	x		x
10	x		x
11	x		x

**Figure 1:**
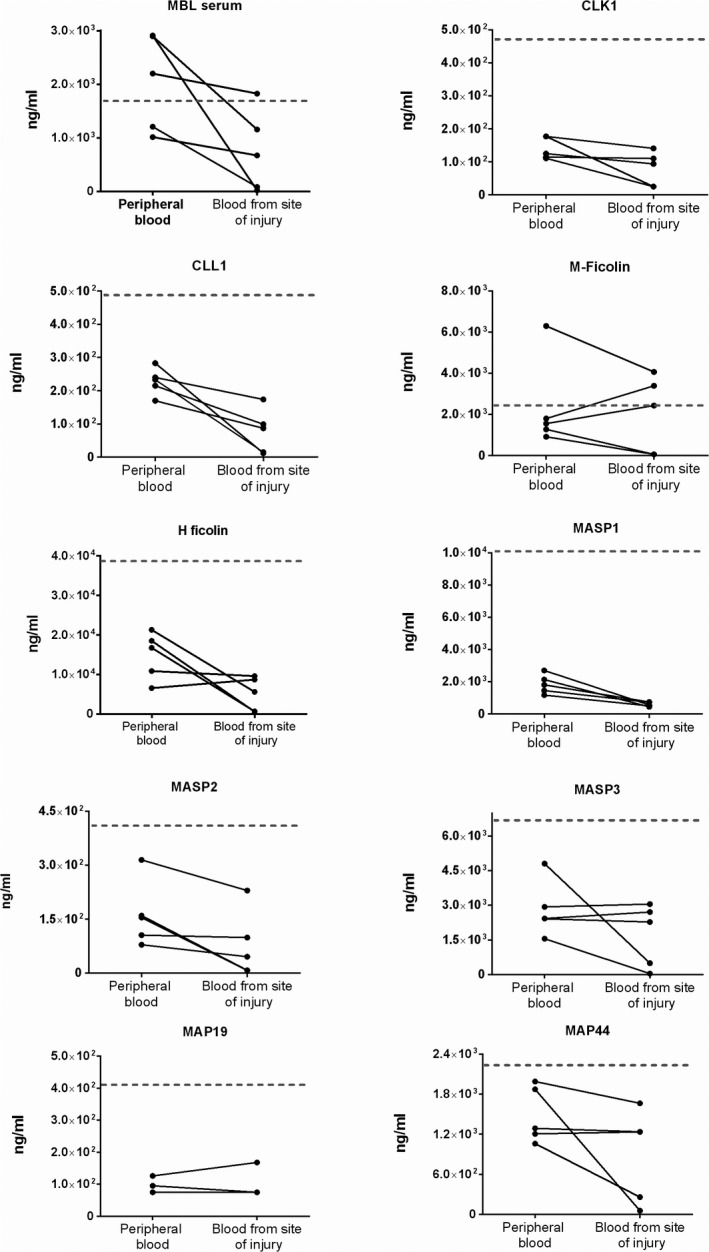
Lectin pathway proteins measured in peripheral blood and in cerebral blood at the site of injury in patients with subarachnoid hemorrhage. Gray horizontal lines indicate median serum concentration of the protein in a group of 300 healthy individuals (Troldborg et al., 2017)

In all patients, the concentration in cerebral blood from the site of injury of MBL, CL‐L1, CL‐K1, MASP‐1, and MASP‐2 was lower than in peripheral blood (Figure 1). Regarding M‐ficolin, H‐ficolin, MAp19, MAp44, and MASP‐3, the results were more ambiguous; the cerebral concentrations were higher in some samples and lower in others compared with peripheral blood (Figure 1).

All measurements, both peripheral and from the site of injury, were lower when measured in SAH patients than the median for controls (gray lines in Figure 1) except for MBL and M‐ficolin.

Proteins of the LP were close to undetectable in CSF and significantly lower than what could be measured in peripheral blood (Table 2).

**Table 2:** Concentration of lectin pathway proteins in patients with subarachnoid hemorrhage, determined in peripheral blood and in cerebrospinal fluid (*n* = 7)

Proteins	Peripheral blood, µg/ml (median + IQR)	Cerebrospinal fluid, µg/ml (median + IQR)
MBL	0.95 (0.79–1.41)	0.03 (0.01–0.06)
H‐ficolin	19.24 (12.29–30.42)	0.40 (0.09–1.5)
M‐ficolin	1.71 (1.41–3.48)	0.03 (0.02–0.25)
MASP−3	3.48 (2.24–4.70)	0.13 (0.08–0.37)
MAp44	1.32 (1.19–1.41)	0.10 (0.04–0.18)
MASP−2	0.26 (0.19–0.55)	0.01 (0.00–0.02)
MASP−1	2.40 (1.85–5.02)	0.10 (0.08–0.17)
MAp19	0.18 (0.13–0.26)	0.02 (0.02–0.02)
CL‐L1	0.23 (0.15–0.34)	0.01 (0.00–0.01
CL‐K1	0.15 (0.12–0.18)	0.01 (0.00–0.01)

Note

Abbreviations: CL‐K1, collectin kidney‐1; CL‐L1, collectin liver 1; IQR, interquartile range; MAp19, MBL‐associated protein of 19 kDa; MAp44, MBL‐associated protein of 44 kDa; MASP‐1, MASP‐2, and MASP‐3, MBL‐associated serine proteases 1, 2, and 3; MBL, mannan‐binding lectin.

## 4 DISCUSSION

In the present study, we demonstrated lower concentrations of MBL, CL‐L1, CL‐K1, MASP‐1, and MASP‐2 when drawing cerebral blood from the site of injury in SAH patients compared with peripheral blood. The concentrations of all LP proteins were very low in CSF compared with peripheral blood.

Previous studies used peripheral blood or CSF to investigate changes in protein or cytokine concentration related to injury or disease, presuming that local injury is reflected in peripheral blood or CSF (Llull et al., 2017). However, no studies have validated this assumption. The present study is, to our knowledge, the first study attempting to address this question.

Mannan‐binding lectin and CL‐K1 bind to apoptotic cells (Henriksen, Brandt, Iyer, Thielens, & Hansen, 2013; Nauta et al., 2003) and initiate the LP pathway through activation of MASP‐1 and MASP‐2. (Merle & Church, 2015) This could explain why lower concentrations of MBL, CL‐K1, CL‐L1, MASP‐1, and MASP‐2 are observed in the cerebral samples. It is possible that the levels reflect a consumption of the proteins at the site of injury as they bind to damaged or dying cells. A clinical trial is currently being conducted to investigate the use of a MASP‐2 antibody to inhibit the LP in traumatic injury patients, (2016) potentially reducing inflammation and secondary brain injury. Our study supports the hypothesis of LP pathway involvement in SAH at the injured site.

Zanier et al. reported increased M‐ficolin and decreased L‐ficolin concentrations, with no changes in H‐ficolin in SAH patients, compared with healthy individuals. (Zanier et al., 2014) The reduced concentration of ficolins in some of our samples might be caused by an accumulation of ficolins in damaged tissue, as hypothesized by Llull et al., (2017) Contrary to the other pattern recognition molecule, M‐ficolin is mainly produced in peripheral blood leukocytes and bone marrow cells. (Kjaer et al., 2013) This could explain why some of our patients have a higher concentration of M‐ficolin in the cerebral samples, as the SAH causes inflammation and recruitment of neutrophils to the site of injury (Pradilla et al., 2010).

The local changes we observed are only to some extend reflected in the peripheral blood when comparing to the average for controls (Troldborg et al., 2017). For MBL and M‐ficolin, this is not the case. Thus, the findings from the present study suggest that peripheral blood sampling does not always reflect local changes at the site of injury and that for some proteins more information could potentially be obtained when investigating pathogenic mechanisms in blood from the site of injury.

Concentrations of LP proteins in CSF were all lower than in peripheral blood, which is in accordance with a recent study (Llull et al., 2017). Although both studies are relatively small, we can conclude that in SAH measurement of LP proteins in CSF provides very little information about the damage or pathogenesis of the disease.

A limitation of the present study is the small sample size, increasing the risk of overlooking a potential difference or overinterpreting results.

## 5 CONCLUSION

The present study demonstrates a difference in the concentrations of LP proteins in peripheral blood compared with cerebral blood samples of SAH patients, particularly with regard to MASP‐1, MASP‐2, MBL, CL‐L1, and CL‐K1. This supports a role for the LP proteins at the site of injury in SAH. Concentrations of LP proteins in CSF were almost unmeasurable, indicating very little potential use of CSF sampling.

The present study is an exploratory study and the first to compare measurements in peripheral blood and blood drawn from the site of injury in SAH patients. The findings suggest that LP protein concentrations measured in peripheral blood do barely reflect local changes, which should be kept in mind when investigating pathogenic mechanisms at local sites of injury.

## CONFLICT OF INTEREST

The authors have no conflicts of interest regarding the present manuscript.

## AUTHOR CONTRIBUTION

Anne Troldborg, Steffen Thiel, and Anne‐Mette Hvas designed the study. Thorkil Anker‐Møller was in charge of collecting blood samples, consent, and handling the blood samples after they were drawn. Thorkil Anker‐Møller handled patient inclusion and clinical assessments supervised by Niels Sunde, who was the neurosurgeon in charge of the clinical part of the project. Thorkil Anker‐Møller performed the laboratory experiments supervised by Steffen Thiel and Anne Thiel. Steffen Thiel developed the assays used in the project. Thorkil Anker‐Møller performed data analysis supervised by Anne Troldborg and Anne‐Mette Hvas. Thorkil Anker‐Møller, Anne Troldborg, and Anne‐Mette Hvas wrote the manuscript, and all authors participated in the editing of the article.

## Data Availability

The data that support the findings of this study are available from the corresponding author upon reasonable request.
